# Supplementary material for: Modeling glioblastoma heterogeneity as a dynamic network of cell states
Source: Mol Syst Biol. 2021 Sep 16;17(9):e10105. doi: 10.15252/msb.202010105 (PMC8444284; doi:10.15252/msb.202010105)
Supplement: Supplementary file 5 — Source Data for Figure 3 [file MSB-17-e10105-s001.zip › Figure3A_sourcedata/GSEA_3065/hallmarks_state2.GseaPreranked.1623416269049/HALLMARK_CHOLESTEROL_HOMEOSTASIS.html]

Details for gene set HALLMARK\_CHOLESTEROL\_HOMEOSTASIS[GSEA]

|  || Dataset | state2 |
| Phenotype | NoPhenotypeAvailable |
| Upregulated in class | na\_neg |
| GeneSet | HALLMARK\_CHOLESTEROL\_HOMEOSTASIS |
| Enrichment Score (ES) | -0.5169928 |
| Normalized Enrichment Score (NES) | -1.8351367 |
| Nominal p-value | 0.0 |
| FDR q-value | 0.0018097528 |
| FWER p-Value | 0.009 |
Table: GSEA Results Summary

  

Fig 1: Enrichment plot: HALLMARK\_CHOLESTEROL\_HOMEOSTASIS      
 Profile of the Running ES Score & Positions of GeneSet Members on the Rank Ordered List

  

| PROBE | GENE SYMBOL | GENE\_TITLE | RANK IN GENE LIST | RANK METRIC SCORE | RUNNING ES | CORE ENRICHMENT || 1 | ACAT2 |  |  | 180 | 0.294 | 0.0353 | No |
| 2 | TMEM97 |  |  | 331 | 0.223 | 0.0606 | No |
| 3 | FABP5 |  |  | 796 | 0.138 | 0.0368 | No |
| 4 | IDI1 |  |  | 878 | 0.130 | 0.0522 | No |
| 5 | FDPS |  |  | 1297 | 0.098 | 0.0258 | No |
| 6 | PMVK |  |  | 1595 | 0.082 | 0.0093 | No |
| 7 | PLSCR1 |  |  | 1853 | 0.070 | -0.0051 | No |
| 8 | ACTG1 |  |  | 2364 | 0.052 | -0.0499 | No |
| 9 | FDFT1 |  |  | 2717 | 0.041 | -0.0798 | No |
| 10 | ANXA5 |  |  | 2812 | 0.039 | -0.0826 | No |
| 11 | STX5 |  |  | 3365 | 0.026 | -0.1366 | No |
| 12 | MVK |  |  | 3449 | 0.025 | -0.1408 | No |
| 13 | ATF5 |  |  | 3510 | 0.024 | -0.1428 | No |
| 14 | MVD |  |  | 3516 | 0.024 | -0.1390 | No |
| 15 | ECH1 |  |  | 4011 | 0.015 | -0.1888 | No |
| 16 | NFIL3 |  |  | 4213 | 0.012 | -0.2081 | No |
| 17 | HMGCS1 |  |  | 4272 | 0.011 | -0.2122 | No |
| 18 | NSDHL |  |  | 4406 | 0.009 | -0.2248 | No |
| 19 | PDK3 |  |  | 4547 | 0.007 | -0.2385 | No |
| 20 | PCYT2 |  |  | 4577 | 0.006 | -0.2405 | No |
| 21 | ETHE1 |  |  | 4663 | 0.005 | -0.2487 | No |
| 22 | ACSS2 |  |  | 4908 | 0.001 | -0.2745 | No |
| 23 | STARD4 |  |  | 5079 | -0.001 | -0.2924 | No |
| 24 | CXCL16 |  |  | 5157 | -0.003 | -0.3002 | No |
| 25 | HSD17B7 |  |  | 5217 | -0.004 | -0.3058 | No |
| 26 | GNAI1 |  |  | 5503 | -0.009 | -0.3346 | No |
| 27 | S100A11 |  |  | 5569 | -0.009 | -0.3398 | No |
| 28 | FASN |  |  | 6119 | -0.019 | -0.3949 | No |
| 29 | CHKA |  |  | 6122 | -0.019 | -0.3917 | No |
| 30 | GPX8 |  |  | 6123 | -0.019 | -0.3882 | No |
| 31 | EBP |  |  | 6195 | -0.020 | -0.3921 | No |
| 32 | ERRFI1 |  |  | 6445 | -0.024 | -0.4141 | No |
| 33 | LGMN |  |  | 6457 | -0.025 | -0.4107 | No |
| 34 | CPEB2 |  |  | 6650 | -0.028 | -0.4260 | No |
| 35 | ATF3 |  |  | 6737 | -0.030 | -0.4295 | No |
| 36 | SEMA3B |  |  | 6759 | -0.031 | -0.4261 | No |
| 37 | PLAUR |  |  | 6906 | -0.034 | -0.4353 | No |
| 38 | CYP51A1 |  |  | 7467 | -0.050 | -0.4858 | No |
| 39 | CTNNB1 |  |  | 7580 | -0.053 | -0.4880 | No |
| 40 | ABCA2 |  |  | 7853 | -0.061 | -0.5056 | Yes |
| 41 | GLDC |  |  | 7897 | -0.063 | -0.4986 | Yes |
| 42 | SC5D |  |  | 7900 | -0.063 | -0.4871 | Yes |
| 43 | TNFRSF12A |  |  | 7931 | -0.064 | -0.4784 | Yes |
| 44 | LPL |  |  | 8087 | -0.071 | -0.4818 | Yes |
| 45 | TP53INP1 |  |  | 8285 | -0.082 | -0.4877 | Yes |
| 46 | LSS |  |  | 8426 | -0.091 | -0.4858 | Yes |
| 47 | GUSB |  |  | 8446 | -0.092 | -0.4708 | Yes |
| 48 | FADS2 |  |  | 8492 | -0.095 | -0.4580 | Yes |
| 49 | SREBF2 |  |  | 8657 | -0.108 | -0.4555 | Yes |
| 50 | ANTXR2 |  |  | 8666 | -0.108 | -0.4363 | Yes |
| 51 | ATXN2 |  |  | 8705 | -0.112 | -0.4197 | Yes |
| 52 | SCD |  |  | 8982 | -0.146 | -0.4221 | Yes |
| 53 | JAG1 |  |  | 8993 | -0.148 | -0.3958 | Yes |
| 54 | DHCR7 |  |  | 9009 | -0.150 | -0.3696 | Yes |
| 55 | LDLR |  |  | 9117 | -0.170 | -0.3495 | Yes |
| 56 | ALCAM |  |  | 9132 | -0.173 | -0.3189 | Yes |
| 57 | HMGCR |  |  | 9194 | -0.187 | -0.2906 | Yes |
| 58 | SQLE |  |  | 9209 | -0.194 | -0.2562 | Yes |
| 59 | PNRC1 |  |  | 9294 | -0.229 | -0.2227 | Yes |
| 60 | LGALS3 |  |  | 9308 | -0.236 | -0.1804 | Yes |
| 61 | CD9 |  |  | 9324 | -0.248 | -0.1360 | Yes |
| 62 | TM7SF2 |  |  | 9330 | -0.252 | -0.0898 | Yes |
| 63 | CLU |  |  | 9430 | -0.547 | 0.0011 | Yes |
Table: GSEA details [plain text format]

  

Fig 2: HALLMARK\_CHOLESTEROL\_HOMEOSTASIS: Random ES distribution      
 Gene set null distribution of ES for **HALLMARK\_CHOLESTEROL\_HOMEOSTASIS**
